# Nondestructive Inspection of Reinforced Concrete Utility Poles with ISOMAP and Random Forest

**DOI:** 10.3390/s18103463

**Published:** 2018-10-15

**Authors:** Saeed Ullah, Minjoong Jeong, Woosang Lee

**Affiliations:** 1 University of Science and Technology (UST), 217 Gajeong-ro, Yuseong-gu, Daejeon 34113, Korea; saeedonline12@gmail.com; 2 Korea Institute of Science and Technology Information (KISTI), 245 Daehak-ro, Yuseong-gu, Daejeon 34141, Korea; 3 Smart C&S Co., Ltd., Yongsan-dong, Yuseong-gu, Daejeon 34141, Korea; smartcs@chol.com

**Keywords:** nondestructive inspection, machine learning, dimensionality reduction, classification, ISOMAP, random forest

## Abstract

Reinforced concrete poles are very popular in transmission lines due to their economic efficiency. However, these poles have structural safety issues in their service terms that are caused by cracks, corrosion, deterioration, and short-circuiting of internal reinforcing steel wires. Therefore, they must be periodically inspected to evaluate their structural safety. There are many methods of performing external inspection after installation at an actual site. However, on-site nondestructive safety inspection of steel reinforcement wires inside poles is very difficult. In this study, we developed an application that classifies the magnetic field signals of multiple channels, as measured from the actual poles. Initially, the signal data were gathered by inserting sensors into the poles, and these data were then used to learn the patterns of safe and damaged features. These features were then processed with the isometric feature mapping (ISOMAP) dimensionality reduction algorithm. Subsequently, the resulting reduced data were processed with a random forest classification algorithm. The proposed method could elucidate whether the internal wires of the poles were broken or not according to actual sensor data. This method can be applied for evaluating the structural integrity of concrete poles in combination with portable devices for signal measurement (under development).

## 1. Introduction

Reinforced concrete poles are commonly used for telephone and electricity transmission. Their greater mechanical strength, cost effectiveness, longer life span (over 50 years), potential to cover longer distances, and better electrical resistance are the key reasons for their widespread usage [1]. Further, reinforced concrete poles constitute an alternative to steel poles because of their higher variability of architectural shapes and comparatively low maintenance costs. When compared to timber poles, concrete poles offer better resistance to hurricanes and are more robust against decay and fire [2]. However, structural safety defects can occur in reinforced concrete poles because of cracks, voids, corrosion, deterioration, and short-circuiting of internal reinforcing steel wires. These problems in reinforced concrete poles are caused by atmospheric exposure, earthquakes, hurricanes, floods, moisture changes, poor construction practices, and various chemical, mechanical, and physical reactions [3,4,5,6]. Structural problems demand more attention than non-structural problems. According to Doukas et al. [7] approximately half of accidents involving reinforced concrete poles are related to structural defects. These defects constitute a major reason for reduced life expectancy, structural strength, and serviceability. Further, structural defects can result in subsequent failure. Moreover, the failure of poles can cause serious problems, such as falling onto people or animals, causing injury (or death), and service disruption. Early detection of such problems can save precious lives, time, and money. Therefore, it is necessary to identify these defects at an early stage for which accurate inspection and monitoring is mandatory on regular basis. Thus, to ensure the structural integrity of utility poles, those already in service poles must be regularly inspected or constantly monitored [8].

Accurate integrity assessment of existing poles is very important, because maintenance and repair costs are increasing rapidly. Hence, there is an increasing demand to develop more accurate, consistent, and reliable non-destructive methods for assessing the condition of in-service poles [2]. Stewart et al. [5] indicated that structural reliability assessments need to be updated at regular intervals through on-site inspections and testing.

For the inspection of reinforced concrete utility poles, traditional methods, such as visual inspection, are still commonly used. However, direct sampling and observation of the structure is very difficult for utility poles [9]. In such situations, Non-Destructive Testing (NDT) methods are applied. Here, NDT is defined as the course of inspecting, testing, or evaluating materials without destroying the structure or serviceability of any part of the system. Examples of NDT methods for structural health monitoring of concrete (with and without reinforcement) materials are visual inspection, rebound hammer, stress-wave methods, ultrasound monitoring, infrared thermography, nuclear methods, X-ray computed tomography, magnetic and electrical methods, penetrability methods, and radar (Radio Detection and Ranging) [9,10,11,12,13,14,15,16,17]. Although many of these methods render better results, they require highly qualified, experienced, and licensed inspectors for the testing and interpretation of the results. Additionally, they require expensive and bulky equipment to carry out complex signal processing. Electrical power is needed in some methods, which can lead to short circuits in reinforced concrete. Moreover, safety concerns during testing are also a problem in many of these methods. Concerning all of the methods listed above, many case studies exist in which different techniques have been combined to improve performance, reduce cost, or address any other limitations of the individual methods [9,18].

Recently, Dackermann et al. [19,20] developed NDT methods with advanced signal processing and machine learning techniques for timber poles, self-compacting concrete poles without steel reinforcement, and generic concrete poles without steel reinforcement. They adopted stress-wave techniques for data gathering, achieving accuracy levels up to 93%. A large number of tactile transducers and accelerometers (along with other devices) were used in these methods. Dackermann et al. [19] applied Principal Component Analysis (PCA) for feature reduction and Support Vector Machines (SVM) for classification of signals to predict pole damage conditions, while Dackermann et al. [20] used Frequency Response Functions (FRFs) and PCA for extracting signal features when capturing single-mode stress waves for condition assessment. It is very difficult to find any works in the literature related to the application of machine learning or data analysis techniques for NDT on reinforced concrete utility poles.

In this paper, we propose a novel NDT method for structural health monitoring of reinforced concrete utility poles that is based on machine learning techniques. To train the machine-learning algorithm, we used data gathered through magnetic sensors at actual site by SMART C&S [21]. Moreover, ISOMAP [22] is applied on the data for feature reduction, and the resulting refined data constitute the input to a random forest classifier [23,24]. In particular, the refined signals are trained with a random forest method and are categorized into safe and crack signals that are based on actual field experiments. Safe signals are those corresponding to non-damaged wires, whereas crack signals correspond to damaged wires. The proposed system is able to predict 97% correct signals among safe and crack signals on unclassified sensor data. We also applied two other well-known machine-learning algorithms on our data: Support Vector Machine (SVM) [25] and decision trees [26]. The detailed comparison of these two algorithms with random forest on our data is described in Section 4.2. All of the algorithms used in this work were written and executed with the Python language machine learning scikit-learn library [27].

The rest of our paper is organized as follows: Section 2 describes the complete experimental setup for data gathering. The proposed system resulting from our research work is explained in Section 3. The experimental results are presented and discussed in Section 4. Finally, the conclusions are presented in Section 5.

## 2. Experimental Setup

In this section, we present the applied data gathering techniques, the used devices, and their specifications. The whole setup for this project is as follows: a magnetic sensing device, a Data Acquisition (DAQ) device, a cable, and a portable computer. To inspect a utility pole, the magnetic sensing device can be inserted into the pole through holes, and the signals can be gathered with the help of the DAQ device. This device can be attached to a portable computer to check the signals with the patterns of our application. Regarding the training of our algorithm, the signals were gathered with the same device and the dataset was manually configured by the field engineers of SMART C&S. They collected signals from 30 reinforced concrete utility poles containing signals from damaged and non-damaged wires and labeled them accordingly. They uninstalled the poles and broke down the concrete cover over steel wires to pull the wires out the concrete. In particular, the field engineers pulled all of the wires out those 30 poles and gathered the signals from each wire. They inserted the sensors into the poles through the first bolt hole nearest to the ground and detected the signals from bottom portion up to the first bolt hole of the pole, because the tendency of defects in wires inside poles is mostly in the bottom portion. The measuring section for signal gathering inside poles was set to a maximum height of 4 m, and the measurement rate was fixed to 0.3 m/s. The diameter of steel wires in all of the poles was from 9 to 12 mm, and there were 16 steel wires (eight tension and eight reinforcing) in each utility pole. The thickness of the concrete cover over the steel wires was from 12 to 24 mm in each pole. The field engineers gathered 101 Hall Effect values for every signal. These values are referred to as “features” in our dataset. The dataset could contain many ambiguous, repeated, and unimportant features. Therefore, we filtered the dataset into meaningful features to be used with a classification algorithm, which is further explained in Section 3.

**Figure 1:**
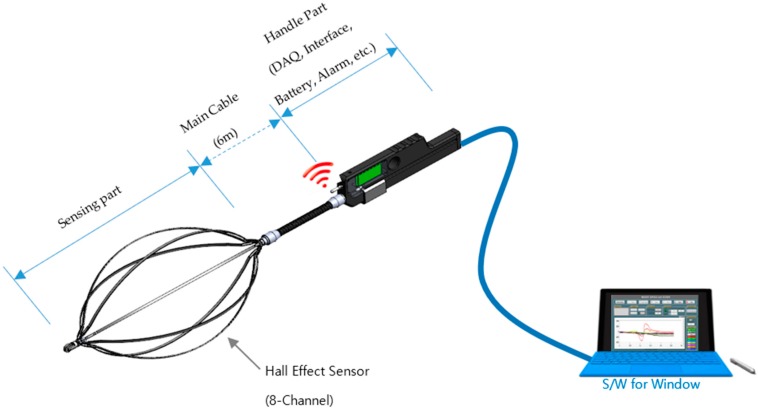
Magnetic sensing device with eight channels.

The eight-channel magnetic sensing device along with all the parts is depicted in Figure 1. The specifications of all the parts of this device are displayed in Table 1. 

**Figure 2:**
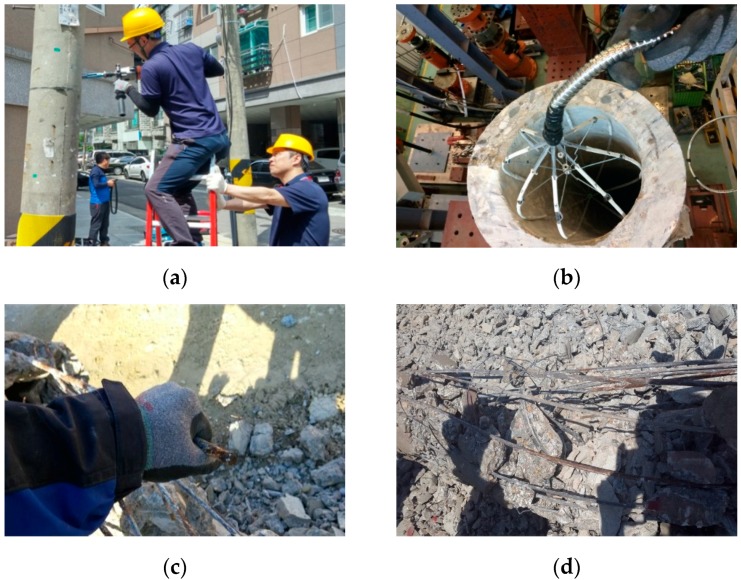
(**a**) Field testing; (**b**) laboratory testing; (**c**) a broken steel wire; (**d**) multiple steel wires.

Figure 2a illustrates the process of removing a maintenance bolt and securing the bolt hole to insert the magnetic sensing device into a working utility pole. Figure 2b shows an example of laboratory verification experiments of the data-gathering device. Figure 2c shows an example of a broken steel wire and Figure 2d shows multiple steel wires inside the poles.

## 3. Proposed System

The dataset for this research project is composed of *n* samples with *m* features, *R* = {(*x_i_^d^*, *y_i_*), *i* = 1, 2, …, *n*}, where in our case *n* = 240, *m* = 101 and *d* = 1, 2, …, *m*, *x_i_* is the input data and every row in *x_i_* has a label *y_i_* ∈ {0, 1}. Note that 0 indicates safe signals, while 1 denotes crack signals. Each row of *x_i_* represents a signal, while *d* indicates the features of every signal, which are the Hall Effect values for each signal.

**Figure 3:**
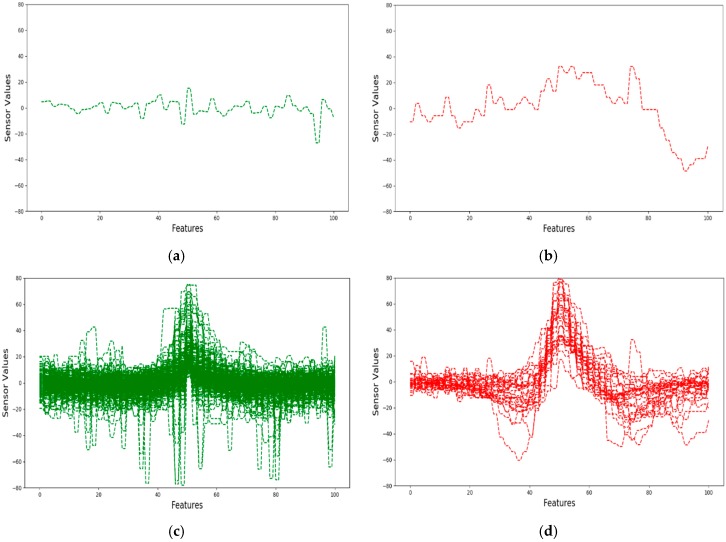
(**a**) A safe signal; (**b**) a crack signal; (**c**) safe signals; and, (**d**) crack signals.

Figure 3 depicts plots of safe and crack signals from our dataset in two-dimensional graphs. Figure 3a shows a single sample of a safe signal in our dataset. Figure 3b depicts a single sample of a crack signal from our dataset. Figure 3c represents all of the safe signals in our dataset. Figure 3d shows all the crack signals in our dataset.

Magnetic sensors were used for data gathering and the dataset contains many replicated features. To reduce these, it is essential to apply a dimensionality reduction technique. Dimensionality reduction is also important for finding meaningful low-dimensional hidden structures in high dimensional data and to improve the performance of classification algorithms.

### 3.1. The Flowchart of Our Proposed System

**Figure 4:**
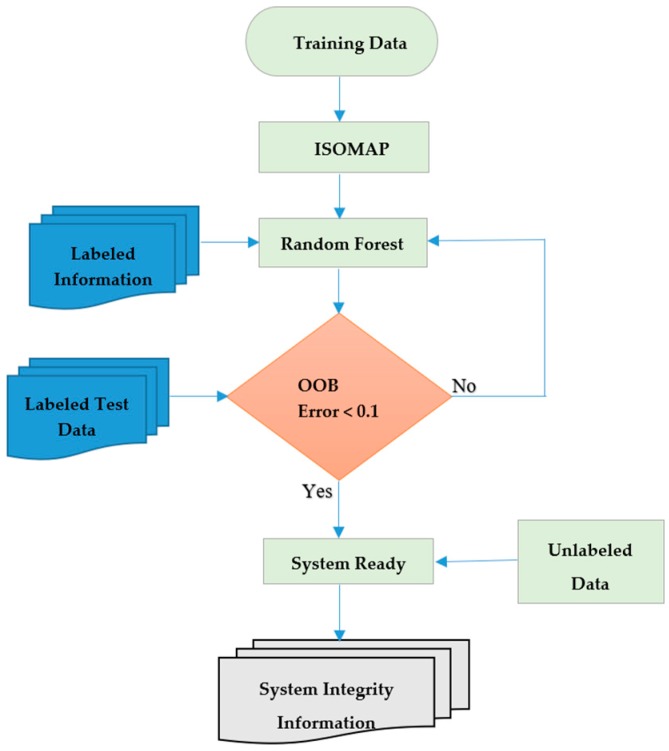
Flowchart of the proposed system.

The overall flow of our proposed system is depicted in Figure 4.

The flowchart in Figure 4 illustrates that for the dimensionality reduction of our data, we applied ISOMAP, which is a manifold-based global geometric framework mainly used for non-linear dimensionality reduction. Non-linear dimensionality reduction techniques became very popular in the last decade due to their superior performance on high dimensional data when compared to linear techniques [28]. Jeong [29] showed that ISOMAP outperforms PCA (a linear dimensionality reduction technique) on high dimensional data.

### 3.2. ISOMAP

ISOMAP is an extension of Multidimensional Scaling (MDS), a classical method for embedding dissimilatory information into a Euclidean space. The main concept of ISOMAP is to replace Euclidean distances with an approximation of the geodesic distances on the manifold. ISOMAP is a three-step process: (1) Construct neighborhood graph; *k*-nearest neighbors of every data point are defined and represented by a graph *G*; in *G*, every point is connected to its nearest neighbors by edges. (2) Compute the shortest paths; the geodesic distances between all the pairs of points are estimated using the Dijkstra algorithm [30]; the squares of these distances are stored in a matrix of graph distances *D*^(*G*)^. (3) Construct d-dimensional embedding; the classical MDS algorithm is applied on *D*^(*G*)^ in order to find a new embedding of the data in a d-dimensional Euclidean space *Y*.

#### Deciding the Number of Dimensions for the ISOMAP Algorithm

Our dataset is composed of 240 data items with 101 features. Firstly, we have to determine the number of dimensions of the ISOMAP space. For this purpose, we calculated the residual variance [22,31], which is typically used to evaluate the error of dimensionality reduction. Residual variance is defined in Equation (1), as follows:

*R_d_* = 1 − *r*^2^ (*G*, *D_d_*) (1)

where *R_d_* is the residual variance, *G* is the geodesic distance matrix, *D_d_* is the Euclidean distance matrix in the d-dimensional space, and *r*(*G*, *D_d_*) denotes the correlation coefficient of *G* and *D_d_*. The value of *d* is determined by a trial-and-error approach to reduce the residual variance.

**Figure 5:**
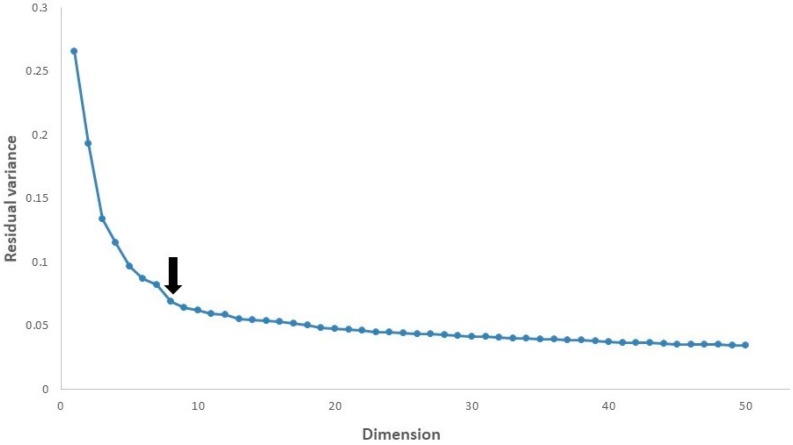
The residual variance of ISOMAP in our data.

Figure 5 shows that in all cases the residual variance decreases as the number of dimensions *d* is increased. It is recommended in [22,31] to select the number of dimensions at which this curve ceases to decrease significantly with added dimensions. To evaluate the intrinsic dimensionality of our data, we searched for the “elbow”, at which this curve is not decreasing significantly with increasing dimensions. The arrow mark highlights the approximate dimensions in our data. If the dimensions are increased and exhibit some residual variance of the increased dimensions, then the data can be better explained by ISOMAP and the classification algorithm will perform better based on the residual variance. For example, in our dataset for a number of dimensions equal to 50 having a residual variance equal to 0.03, the performance is slightly better than for a number of dimensions equal to 8. We set eight dimensions for our classification algorithm because of the computational cost of both ISOMAP and the random forest. We had already achieved better performance with eight dimensions. However, the performance is not improved with increasing dimensions once the residual variance becomes zero.

### 3.3. Random Forest

ISOMAP reduces our dataset into eight dimensions on which a random forest classification algorithm is applied for the purpose of training features in the data. The random forest is an ensemble method, and generally, ensemble methods perform better than single classifiers. They are built from a set of classifiers and then use the weightage of their predictions to render the final output [32]. These methods employ more than one classification technique and then combine their results. They are notably less prone to overfitting. The random forest algorithm consists of three steps: (1) Construct *n*_trees_ bootstrap samples from the input data while using the Classification And Regression Trees (CART) [33] methodology. (2) Grow an unpruned tree for each of the *n*_trees_, randomly sample *m*_try_ of the predictors, and choose the best split among those variables. (3) Aggregate the predictions of the *n*_tree_ trees for the prediction of new data. We applied the scikit-learn [27] implementation that combines classifiers by averaging their probabilistic prediction.

Choosing the number of trees in a random forest is an open question. Breiman [23] mentioned that, the greater the number of trees, the better the performance of the random forest. However, it is very difficult to find the optimal number of trees for the algorithm. Oshiro et al. [34] applied random forest on 29 different types of datasets with varying number of trees *L* in exponential rates using base two, which is *L* = 2*^j^*, *j* = 1, 2, …, 12. They concluded that sometimes a larger number of trees in the forest only increases its computational cost, having no significant impact on its performance. We applied the same method as that of Oshiro et al. [34] to decide the number of trees in our random forest classifier. The number of trees and their performance on our dataset is reported in Table 2.

Table 2 shows that using 8, 16, 32, 64, or 128 as the number of trees in a random forest on our dataset resulted in the same performance. Therefore, we set the number of trees to eight. We did not set a large number of trees, as this would increase the computational cost.

To decide on the number of random samples, the common term applied is $\sqrt{p}$, where *p* is the number of predictors. Our dataset for random forest has eight features, therefore, we choose *m*_try_ = 3.

The OOB (out-of-bag) error estimate is used to check the performance of a random forest model. This error is calculated by averaging only those trees corresponding to bootstrap samples that render wrong predictions [23,35]. We stopped training our model when the OOB error reduced below 10%, which is a quite good fit and shows that more than 90% of the decision trees are rendering correct predications. As we are applying random training to a random forest, we executed the model 100 times and then calculated the mean OOB error from all the OOB error values.

## 4. Results and Discussion

### 4.1. Performance Evaluation of Our Classifier

We randomly chose 210 data items for training and 30 data items to test our classification algorithm. The structural safety labels were separated and were not shown to the algorithm for evaluation purposes. The predicted results from the algorithm were then compared with the labels. The accuracy was calculated as the ratio between accurate predicted instances and the total number of examined instances, as defined in Equation (2). As the random forest algorithm renders different results on different executions, it was executed 100 times. The accuracy, defined as the mean value of all the 100 calculated accuracies, was 97% in our case. Our dataset consisted of 211 safe signals and 29 crack signals. Therefore, it is an imbalanced dataset. It is likely that the algorithm will only predict safe signals and calculate better accuracy on the basis of the result. In such cases, accuracy alone is not enough for determining the performance of an algorithm. The accuracy mostly biases to the majority class data, and presents several other weaknesses, such as less distinctiveness, discriminability, and informativeness. Further, it is possible for a classifier to perform well on one metric while being suboptimal on other metrics. Moreover, different performance metrics measure different tradeoffs in the predictions made by a classifier. Therefore, it is essential to assess algorithms on a set of performance metrics [36]. We thus calculated the confusion matrix, precision, recall [37], and F-measure [37,38], to discriminate accurately and to select an optimal solution for our model. The confusion matrix is widely used for describing the performance of a classifier, because it displays the ways in which a classification model is confused during predication. The confusion matrix is composed of rows and columns corresponding to the classification labels. The rows of the table represent the actual class, while the columns represent the predicted class. The main diagonal elements in the confusion matrix are TP and TN, which denote correctly classified instances, while other elements (FP and FN) denote incorrectly classified instances. Here, TP means true positive, which is when the algorithm correctly predicts the positive class, while TN means true negative, which is when the algorithm correctly predicts the negative class. Further, FP means false positive, which is when the algorithm fails to predict the negative class correctly. Finally, FN means false negative, which is when the algorithm fails to predict the positive class correctly. In our data, the safe signals are represented as a positive class, while crack signals are represented as a negative class. The confusion matrix of our random forest algorithm on the test data is presented in Table 3.

Note that total test points are equal to 30, and the total number of correctly classified instances is calculated as 25 + 4 = 29, while the total number of incorrectly classified instances is given by 0 + 1 = 1.

The confusion matrix clearly shows that there is only one mistake made by our algorithm, namely a crack signal predicted as safe signal. On the basis of the confusion matrix, the result is quite good. However, an analysis exclusively based on the confusion matrix is not sufficient when evaluating the performance of an algorithm. In the case of imbalanced classes, precision, and recall constitute a useful measure of success of prediction. Precision and recall are commonly used in information retrieval for evaluation of retrieval performance [39]. Precision is applied to measure the correctly predicted positive patterns from the total predicted patterns in a positive class, whereas recall is used to find the effectiveness of a classifier in identifying positive patterns. For full evaluation of the effectiveness of a model, examining both precision and recall is necessary. High precision represents a low false positive rate, while high recall represents a low false negative rate. Accuracy *A*, precision *P*, and recall *R* are defined in Equation (2).

(2) $$A=\frac{TP+TN}{TP+FP+FN+TN}\;,\;P=\frac{TP}{TP+FP}\;\mathrm{and}\;R=\frac{TP}{TP+FN}$$

The precision of our classifier on the test data is 96%, while the recall is 100%. These are high values. However, precision and recall are still not sufficient to select an optimal solution or algorithm. To find a balance between precision and recall, F-measure is used. For computing score, the F-measure considers both precision and recall. Moreover, F-measure indicates how many instances the classifier classifies correctly without missing a significant number of instances. The greater the F-measure, the better the performance of the model. F-measure is defined as the harmonic mean of precision and recall, as expressed in Equation (3).

(3) $$F-\mathrm{measure}=\frac{2}{1/P+1/R}$$

The F-measure of our algorithm is 98%. The performance can now be measured in terms of classification accuracy, confusion matrix, precision, recall, and F-measure. We calculated all of them for the evaluation of our classifier, and the results demonstrate that our classifier is very reliable.

### 4.2. Comparison of Random forest with SVM and Decision Trees on Our Data

**Figure 6:**
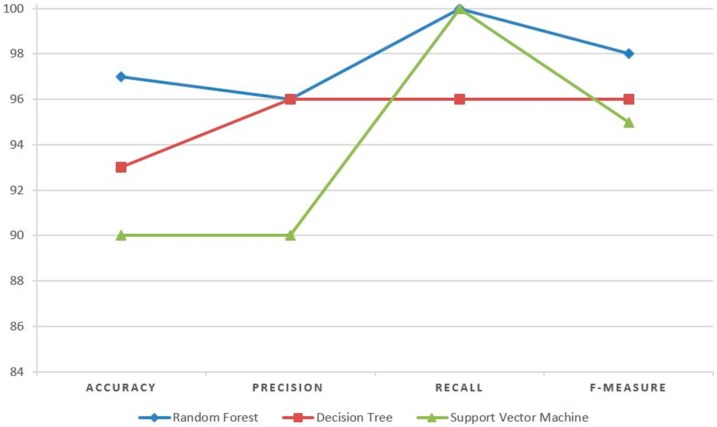
Performance measure graph of different algorithms.

To evaluate and compare the results of a random forest with other machine learning algorithms on our ISOMAP-based reduced data, we applied SVM and decision trees. SVM attempts to find the optimal separating hyperplane between objects of different classes. Further, SVM is the most suitable for binary classification but can also be configured for multi-classification tasks. Decision trees are applied for both classification and regression tasks. In decision trees, each node represents a feature, each link represents a decision, and each leaf represents an outcome. The root node is defined as the attribute that best classifies the training data. This process is repeated for each branch of the tree. An SVM classifier with sigmoid kernel and a gini index-based unpruned decision tree with best split method were implemented. Using SVM and decision trees, we achieved 90% and 93% accuracy, respectively. A complete comparison of all these algorithms on our test data is depicted in Figure 6.

Figure 6 clearly shows that random forests outperform SVM and decision trees. The precision of random forests and decision trees are the same, as well as the recall of random forests and SVMs, but the accuracy and F-measure of random forests are superior.

## 5. Conclusions

In this paper, we proposed a structural safety assessment method for reinforced concrete utility poles with the use of ISOMAP and random forests. The proposed system is able to identify the condition of wires inside reinforced concrete poles. It is also an easy and inexpensive system to adopt, because only a few devices are needed. We had a limited number of trained data items from field experiments. Therefore, we opted for machine learning techniques rather than deep learning methods. We used ISOMAP for data reduction and then applied a random forest classifier for classification purposes. The random forest algorithm outperformed other machine learning algorithms (such as SVM and decision trees) on our data set. In our first attempt, we achieved better performance. In future, we can receive more experimental data from field engineers and apply deep learning methods to accomplish better performance with higher reliability.

## Figures and Tables

**Table 1:** Specifications of all the parts of the magnetic sensing device.

Sensor and DAQ	
ADC Resolution	16 bit
ADC Input Channel	8 Differential Input Channels
ADC Sampling rate	50 S/s
**Main Cable**	
Length	6 m
Diameter	22 mm

**Table 2:** Performance evaluation with different number of trees.

**No of Trees**	2	4	8	16	32	64	128	256	512
**Accuracy**	0.93	0.94	0.97	0.97	0.97	0.97	0.97	0.98	0.98

**Table 3:** Confusion matrix of our proposed system.

	Predicated Safe Signals	Predicated Crack Signals
**Actual Safe Signals**	TP = 25	FN = 0
**Actual Crack Signals**	FP = 1	TN = 4
